# Severe Desquamating Disorder After Liver Transplant: Toxic Epidermal Necrolyis or Graft Versus Host Disease?

**Published:** 2006-01-28

**Authors:** John T. Schulz, Robert L. Sheridan

**Affiliations:** Bridgeport Hospital, Bridgeport, CT; Yale New Haven Health System, New Haven, CT; and Massachusetts General Hospital, Boston, MA

## Abstract

**Objective:** The objective of this article is to present and discuss the clinical characteristics of TEN (Toxic Epidermal Necrolysis) and GVHD (Graft Versus Host Disease) following orthotopic liver transplantation. **Methods:** Recent experience with a patient who suffered a fatal desquamation syndrome within weeks of liver transplantation provides the basis for a discussion of differential diagnosis of these two conditions. **Results:** TEN and GVHD demonstrate similar clinical presentations as well as etiology (CD8+ lymphocyte attack on epithelial surfaces). This case demonstrates the difficulty in distinguishing between these two conditions in this particular patient. **Conclusions:** Advances in the understanding and treatment of one of these disease states may provide therapeutic insights into the other.

We recently cared for a patient who suffered a fatal desquamation syndrome within a few weeks of orthotopic liver transplantation. Although the patient's working diagnosis was toxic epidermal necrolysis (TEN), his history of recent liver transplant suggested the possibility of another diagnosis: graft versus host disease (GVHD). While TEN is well known to burn care providers, GVHD is something that many have never encountered. Here we present the case, followed by a discussion of the clinical characteristics of both TEN and GVHD following orthotopic liver transplantation.

## CASE REPORT

A 55-year-old man presented to our hospital complaining of fever, rash, and a sore mouth. Twenty-three days prior the patient had undergone orthotopic liver transplantation for chronic active hepatitis C with a T_1_N_0_M_0_ hepatocellular carcinoma. His transplantation was without immediate complication, and he was discharged on the following new medications: bactrim ss daily, aspirin 325 mg daily, azothioprine 100 mg daily, tacrolimus 2 mg daily, and valgancyclovir HCl 900 mg daily. The patient had been treated with antithymocyte globulin during induction of immunosuppression.

The patient did well until 17 days after transplant when he presented with fever. Abdominopelvic computed tomography demonstrated a hepatic subcapsular hematoma, and he was treated with piperacillin/tazobactam for a presumed infected hematoma. He was discharged on posttransplant day 21 on oral ampicillin/sulbactam. Shortly after discharge, he developed diffuse erythroderma, fever, and a sore mouth and returned to the hospital. The burn service was consulted.

The patient was ill-appearing with an oral temperature of 104°F, a heart rate of 122, a blood pressure of 120/64 mm Hg, and a respiratory rate of 24; he had widespread macular erythema and a few hemorrhagic bullae, as well as oropharyngeal ulceration. Nikolsky's sign was positive in multiple locations. He was admitted to the burn intensive care unit and topical antimicrobials were applied to his wounds.

The patient had no known drug allergies. His past history was notable only for type 2 diabetes mellitus and ethanol dependence (abstinent for 1 year). He was taking no alternative medicines. The patient's laboratory results were notable for a serum creatinine level of 1.4 mg/dL (increased over a normal baseline) and a white blood cell count of 0.1 with a differential showing no neutrophils. A skin biopsy revealed full-thickness epithelial necrosis, consistent with a diagnosis of Stevens-Johnson syndrome or TEN. There was a sparse lymphocytic infiltrate in the papillary dermis.

Although his airway was initially stable, within 24 hours of admission he was intubated for airway protection as his oropharyngeal mucosa began to disintegrate. Meanwhile, his bullous erythroderma progressed until most of his epidermis had sloughed off and he developed intermittent atrial fibrillation and a vasopressor requirement. In consultation with infectious disease medicine and transplant surgery services, the patient was begun on empiric vancomycin and aztreonam and all of his immunosuppressants were held except for tacrolimus (dose adjusted down).

Over the next several days, the patient's condition continued to decline with anuric renal failure and Staphylococcal/Candidal septic shock. Filgrastim failed to evoke an increase in his circulating neutrophils. He became progressively hypothermic and his septic shock worsened. An ultrasound of the hepatic artery showed that it was patent. Continuous renal replacement therapy was started 4 days after admission. The patient gradually became increasingly acidotic and hypotensive and died 7 days after admission. The patient was given human intravenous immunoglobulin (IVIG) during his course because of a very low measured serum IgG level. At the request of the transplant surgery service, he also received a short course of high-dose solumedrol. Among candidate etiologic agents, sulfa and beta-lactam antibiotics stood out: both of these had been started within the 3 weeks prior to the patient's presentation with TEN and both are known to be frequently implicated in TEN.^1^,^2^

## DISCUSSION

This patient's diagnosis may well have been TEN, and if so, this constitutes the first case report of severe TEN following liver transplant in the United States. There are 2 previous reports of TEN following liver transplant, one in the French literature^3^ and the other involving a case of mild desquamation without mucous membrane involvement.^4^ Because posttransplant GVHD bears such a strong clinical resemblance to TEN, GVHD is a diagnosis that must be maintained in the differential for all of these patients. Below, we review the clinical characteristics of both TEN and GVHD, illustrating the diagnostic ambiguity in our patient's case.

Toxic epidermal necrolysis was first recognized as a distinct clinical entity approximately 50 years ago.^5^ It is characterized by what appears to be an autoimmune attack on the patient's epithelial surfaces within a few weeks of exposure to a new medication. By definition, at least 30% of the total body surface area of the skin is involved. Other commonly stricken surfaces are the conjunctivae and mucosae (airway, enteric, genitourinary). The loss of skin and mucosal barriers leaves the patients prey to infection. Airway complications are common and intubation for airway protection is often required. In its most severe manifestation, TEN appears to cause bone marrow depression with resulting leukopenia, anemia, and thrombocytopenia. Death in the TEN patient is usually a result of septic shock/multiple organ failure.^6^

The epidemiology of TEN is reasonably uniform worldwide, striking about 1 person per million per year. The syndrome is deadly, with the mortality rate ranging from 30% to 60% depending on the population under study: the older patient, the patient with more comorbidities, and the patient with a septic complication is more likely to die.^7^ A prognostic multiparametric scoring system called SCORTEN was recently elaborated by Bastuji-Garin and colleagues.^8^

Toxic epidermal necrolysis is preceded by toxin (medication) exposure, usually a new medication that the patient has been taking for less than 1 month. Common culprits are the nonsteroidal anti-inflammatory drugs, sulfa and beta-lactam antibiotics, and anticonvulsants, although there are case reports of hundreds of different medication exposures (even steroids). External beam radiation therapy given with anticonvulsants appears to increase the risk of TEN by 2 to 3 orders of magnitude^9^ as does antiviral use by patients infected with HIV.^10^

In the past several years, a link between the Fas ligand and Fas ligand receptor and TEN has been posited.^11^,^12^ This hypothesis led to the introduction of IVIG (an inhibitor of Fas ligand binding) as a treatment for TEN, and several series and case reports supporting the utility of IVIG treatment have been published.^13^,^14^ None of these is convincing, and there is equally good evidence that IVIG does not appreciably affect the mortality of the disease.^15^,^16^ Recent studies using lymphocytes derived from the blister fluid of TEN patients suggest that the disease is mediated by CD8+ cytotoxic T cells that kill their epithelial cell targets by the perforin/granzyme mechanism^17^ and that the endangered basal keratinocytes actually elaborate the Fas ligand in order to destroy the attacking CD8+ cells. If these data prove repeatable, they may explain the clinical inadequacy of IVIG treatment. Corticosteroids, another treatment used in the past, probably do not alter the progression of TEN and increase the risk of infection.^18^,^19^ The current standard of care for TEN is supportive.

Lack of a good animal model for TEN^20^ and the rarity of the syndrome have hampered investigation of its etiology at the cellular level, but, as noted above, the syndrome appears to be mediated by cytotoxic (CD8+) thymocytes. Consequently, the case reported here is surprising. The patient had just had a liver transplant and had received antithymocyte globulin as part of his immunosuppressive induction. Following the transplant, he was taking 3 immunosuppressive medications. Nonetheless, within 3 weeks of starting Bactrim pneumocystis prophylaxis and within 1 week of starting beta-lactam antibiotics he developed an illness indistinguishable clinically from TEN.

Reviewing the patient's laboratory studies after his death revealed that his lymphocyte count began to increase about 3 weeks after his transplant and continued to increase as a percentage of his total leukocytes even as his white blood cell count plummeted. At the time he was most neutropenic, all of his circulating leukocytes were lymphocytes, consistent with the hypothesis that his disease was CD8+ thymocyte mediated. We have no evidence indicating whether the patient's circulating CD8+ thymocytes were donor or recipient lymphocytes. If the lymphocytes were donor derived, then the diagnosis of GVHD would be more likely than TEN:

1. Although GVHD is commonly associated with marrow (or stem cell) transplants,^21^ it is a known rare complication of orthotopic liver transplantation with an incidence of somewhere between 0.1% and 1%. A liver transplant transfers up to 1 billion donor leukocytes (T cells, monocyctes, natural killer cells) to the recipient.
2. Risk is significantly increased when the recipient and the donor share a major histocompatibility antigen haplotype,^22^ making the incidence of GVHD higher in living-related donor liver transplants. Additional risk factors appear to be recipient age older than 65 years and a 40-year age difference between a younger donor and an older recipient.
3. GVHD usually occurs a few weeks (range 1–8 weeks) after transplant.
4. Initial symptoms are usually fever, diarrhea, and an erythematous macular rash.
5. Treatments have ranged from increased immunosuppression (to incapacitate the donor lymphocytes: steroids and antithymocyte globulin) to withdrawal of immunosuppression (to allow recipient lymphocytes to kill donor lymphocytes). No treatment has been proven effective and survival is poor (20% at most).

Most, but not all, cases of post–liver transplant GVHD are accompanied by diarrhea secondary to cytotoxic T-cell attack on the gastrointestinal mucosa. TEN is also often accompanied by immune destruction of gut epithelium. Our patient did not present with diarrhea. In fact, one of the notable aspects of his course was that he tolerated tube feeds well and continued to have bowel movements until the last 2 days before he died. Demonstration of macrochimerism (significant number of donor lymphocytes) in circulating lymphocytes is necessary for a conclusive diagnosis of GVHD. These data were not available for our patient postmortem as the family declined autopsy. In the absence of such data, it is impossible to differentiate between the diagnoses of TEN and GVHD in the weeks following orthotopic liver transplantation. Unfortunately, correctly distinguishing between these 2 clinical entities currently offers no advantage to the patient: there are no proven specific therapies for either syndrome except the provision of supportive critical care. Future work may, however, produce a specific therapy for either TEN or GVHD. Given their similar clinical presentation and their origin in CD8+ attack on epithelial surfaces, advances in the treatment of one of these may give therapeutic insights into the other.
